# Genetic polymorphisms and ovarian cancer risk and response to paclitaxel/cisplatin chemotherapy

**DOI:** 10.1186/1897-4287-13-S1-A4

**Published:** 2015-09-09

**Authors:** Karolina Tecza, Jolanta Pamula-Pilat, Zofia Kolosza, Natalia Radlak, Ewa Grzybowska

**Affiliations:** 1 Center for Translational Research and Molecular Biology of Cancer, Maria Sklodowska-Curie Memorial Cancer Center and Institute of Oncology, Gliwice Branch, Gliwice, Poland; 2 Department of Epidemiology and Silesia Cancer Registry, Maria Sklodowska-Curie Memorial Cancer Center and Institute of Oncology, Gliwice Branch, Gliwice, Poland; 3 Institute of Automatic Control, Silesian University of Technology, Gliwice, Poland

## 

Single nucleotide polymorphisms modulate the risk of developing ovarian cancer during lifetime. In this study we analyzed 12 polymorphic variants and 2 deletions in *PGR, ABCB1, ABCG2, GSTT1, GSTM1, GSTP1, ATM, TP53* and *ATP7B* genes. Ten genetic modifications were significantly associated with the risk of developing ovarian carcinoma in at least one of the groups under study. *PGR* gene polymorphisms’ impact on ovarian cancer risk was specific only for the group of the *BRCA1* mutation carriers, which proves the difference in the modulation of ovarian cancer risk between sporadic and hereditary malignancies, including the breast-ovarian cancer group (as a cancer-prone group). The analyses showed also the importance of *ATP7B* gene in ovarian carcinogenesis, both studied variants of which significantly modulated the ovarian cancer risk in three out of four groups. Cumulative risk analysis revealed 3 unfavorable variants that significantly increased the risk of developing ovarian cancer, and also two favorable genotypes which protected against ovarian cancer.

Survival analysis for carriers of favorable versus unfavorable genotypes emphasized the importance of regulation of the cell cycle and active transport of xenobiotics during paclitaxel/cisplatin chemotherapy. The unfavorable variants could facilitate carcinogenic process and once their carriers developed malignancy, their chances of survival were smaller. Our analyses also showed a strong gene-dosage effect with the decrease of progression-free survival for the carriers of two unfavorable genetic factors.

